# Effect of Diagnostic and Treatment Delay on the Risk of Tuberculosis Transmission in Shenzhen, China: An Observational Cohort Study, 1993–2010

**DOI:** 10.1371/journal.pone.0067516

**Published:** 2013-06-27

**Authors:** Shiming Cheng, Wei Chen, Yingzhou Yang, Ping Chu, Xiaoli Liu, Meigui Zhao, Weiguo Tan, Li Xu, Qingfang Wu, Hongyun Guan, Jinhong Liu, Haitao Liu, Ray Y. Chen, Zhongwei Jia

**Affiliations:** 1 National Centre for Tuberculosis Control and Prevention, Chinese Center for Disease Control and Prevention, Beijing, China; 2 Shenzhen Chronic Disease Control Centre. Shenzhen, China; 3 Key Laboratory of Major Diseases in Children and National Key Discipline of Pediatrics, Ministry of Education, Beijing Pediatric Research Institute, Beijing Children’s Hospital, Capital Medical University, Beijing, China; 4 Bureau of Disease Control and Prevention, Ministry of Health, Beijing, China; 5 National Institute of Allergy and Infectious Diseases, U.S. National Institutes of Health, based at the U.S. Embassy, Beijing, China; 6 National Institute on Drug Dependence, Health Science Centre, Peking University, Beijing, China; 7 Takemi Program in International Health, Department of Global Health and Population, Harvard School of Public Health, Boston, Massachusetts, United States of America; Johns Hopkins Bloomberg School of Public Health, United States of America

## Abstract

**Introduction:**

To understand better the risk of tuberculosis transmission with increasing delay in tuberculosis treatment, we undertook a retrospective cohort study in Shenzhen, China.

**Methods:**

All pulmonary tuberculosis cases in the Shenzhen tuberculosis surveillance database from 1993–2010 were included. Sputum smear positivity and presence of pulmonary cavity were used as proxies for risk of tuberculosis transmission.

**Results:**

Among 48,441pulmonary tuberculosis cases, 70% presented with symptoms of pulmonary TB, 62% were sputum smear positive, and 21% had a pulmonary cavity on chest x-ray. 95.3% of patients self-presented for evaluation of illness after a median 58 days of delay after symptoms began. The proportion presenting sputum smear positive (p<0.001) and with a pulmonary cavity (p<0.001) increased significantly with increasing duration of delay.

**Conclusions:**

Delayed diagnosis and treatment of tuberculosis is associated with a significantly increased risk of pulmonary sputum smear positivity and pulmonary cavity. To decrease risk of transmission, treatment delay needs to be reduced further.

## Introduction

Delays in tuberculosis (TB) treatment have been reported to be a primary source of ongoing TB transmission because of the prolonged infectious period [1]–[3]. The World Health Organization (WHO) reported that China has made significant progress in TB control and prevention in the past decade, with prevalence being halved and mortality reduced by 78%, suggesting that case detection has improved in China as well [4]. As new diagnostic tools are introduced, treatment delay can be reduced, leading to earlier therapy and interruption of disease transmission [5]–[7]. Despite this, TB patients in China are generally identified passively when patients self-present with illness rather than through screening actively and therefore are generally not treated until illness [8].

Risk factors associated with a delay in the diagnosis and treatment of TB have been identified [1]–[3], [9], [10], but the impact of this treatment delay on TB transmission in the community, to our knowledge, has not been well studied in China. In this study, we aim to estimate the effect of treatment delay on the risk of transmission of TB. Data from this kind of study are important for TB policy making on an international scale [11].

## Materials and Methods

### Ethics Statement

The data for this study was provided by “Shenzhen Chronic Disease Control Centre” which is responsible for surveillance TB epidemic in Shenzhen. This study used data collected during routine disease control surveillance and, therefore, ethics committee approval and written consent were not sought [12]. All patients provided informed consent prior to being added to the database. All data was analyzed anonymously.

### Setting

**Figure 1:**
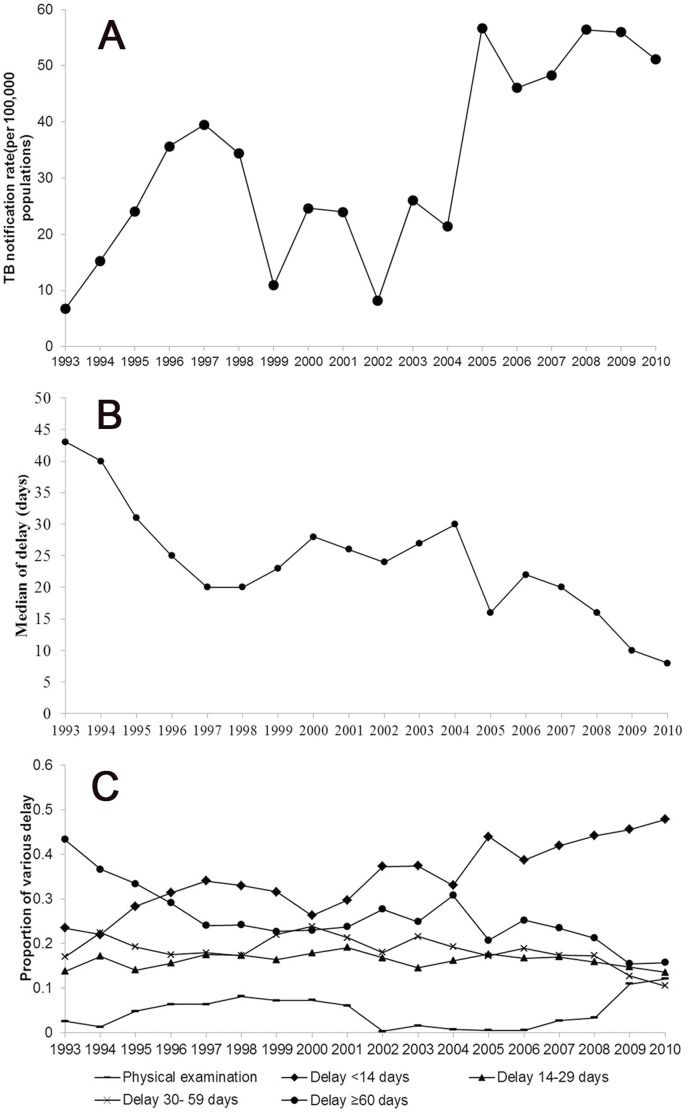
A. The trend of TB notification rate during 1993–2010. B. Overall treatment delay trend over time stratified from 1993–2010. C. Proportion of different treatment delay durations over time from 1993–2010.

Shenzhen, a city bordering Hong Kong, covers 2020 square kilometers and has a total population of 18 million, 80% of whom are migrants, now known as the “migrant civilization city”. Migrants were defined as people who reside Shenzhen but leave “hukou” in their hometown [13], [14]. Shenzhen was originally established 30 years ago as a special economic zone and “the window of China to the outside world”, with more progressive economic policies than the rest of China and consequently attracts workers from across the country [15]. With so many people constantly moving in and out of the city, TB control is quite challenging. Shenzhen pioneered enhanced TB control through DOTS beginning in1993, utilizing funds from the World Bank and the local government [16]. Currently, DOTS coverage approaches 100% and free diagnosis and treatment have been offered to all TB patients, whether permanent resident or migrant, since 2002 [17], [18]. The trend of TB notification rate during 1993–2010 is shown in the Figure 1A.

### Study Design and Patients

We conducted a retrospective cohort study of tuberculosis patients in the Shenzhen surveillance database to identify the role of treatment delay on risk of TB transmission and determine the risk factors associated with treatment delay. Patients were stratified by how they were identified. Physical examination patients were identified during routine physical examination and did not self-present to the healthcare system for evaluation of illness. Employers nationwide provide a general medical examination to their employees, which is not for specific for TB. Treatment delay patients self-presented to the healthcare system for medical evaluation of illness and were diagnosed with TB. Treatment delay duration was defined as the time interval from onset of symptoms (by patient recall) to initiation of treatment and was stratified into four categories : <14 days, 14–29 days, 30–59 days and ≥60 days.

All pulmonary TB cases in the Shenzhen surveillance database, a component of the National TB Surveillance System (NTBSS), from Jan 1, 1993, to Dec 31, 2010 were included. Cases were diagnosed according to standards issued by the Ministry of Health of China, the latest in 2003 [18]. For a diagnosis of sputum smear-positive TB, a case must meet one of the following : (a) 2 positive sputum smears by microscopy, (b) 1 positive sputum smear and 1 positive sputum culture, or (c) 1 positive sputum smear with typical pathology of active TB on chest X-ray [19]. Data collected included gender, age, residency status (permanent/migrant), symptoms (only respiratory symptoms, only constitutional symptoms, or both), pulmonary cavity by chest x-ray (yes/no), and treatment delay duration by patient recall (physical examination, <14 days, 14–29 days, 30–59 days, and ≥60 days). Respiratory symptoms included dyspnea, hemoptysis, cough, or expectoration while constitutional symptoms included malaise, loss of appetite, weight loss, fatigue, fever, chills, chest pain or night sweats.

### Statistical Analysis

We compared the proportion of smear positive tuberculosis and presence of pulmonary cavity with the duration of treatment delay, using a multivariate 3-level logistic regression model adjusted for clustering among the six regions of Shenzhen, demographics (male vs. female) and residence status (migrant vs. permanent resident). We were not able to measure the actual risk of TB transmission because this study is retrospective. Instead, we used sputum smear positivity and presence of pulmonary cavity as proxy measures for risk of TB transmission [20], [21].

The observation was repeated and crossed different regions, we examined the risk factors for each of these two variables by a multilevel multivariate logistic regression analysis. In the model, the region was fitted as 3-level in order to detect whether the two proxies varied cross the regions, observation year as 2 level and each individual as 1 level. Risk factors related with delay were evaluated by a full multivariate linear regression model in which delay was a continue variable and region, year, age, gender, baseline symptoms and migrant status were fitted. We also examined the proportion of treatment delay over time to understand better delay trends from 1993–2010.

By convention, we took p-values <0.05 to indicate significance. All statistical analysis was done with MLwiN 2.02 (Multilevel Models Project Institute of Education).

## Results

The study included 48,441pulmonary tuberculosis patients, among whom median age was 27 years, 63% were male, and 78% were migrants (Table 1). Overall, 70% presented with symptoms of pulmonary TB, 62% were sputum smear positive, and 21% had a pulmonary cavity on chest x-ray identified at presentation. Among the 2261 (4.7%) patients identified by routine physical examination, 11% already had symptoms of pulmonary TB, 31% were already sputum smear positive, and 5% already had a pulmonary cavity on chest x-ray. The remaining 95.3% of patients self-presented to the healthcare system for medical evaluation of illness and were diagnosed with TB after a median duration of delay of 18 days. The proportion presenting sputum smear positive and with a pulmonary cavity increased significantly with increasing duration of delay (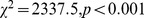) for sputum smear positive; (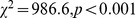) for pulmonary cavity positive).Yearly delay decreased over time from 1993–2010 (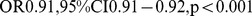). Overall median duration of treatment delay declined significantly from 1993–2010 (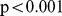, Figure 1B). The proportion of tuberculosis patients identified by physical examination remained stable from 1993–2008, then increased in 2009–2010 (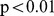, Figure 1C), with the decline primarily due to the decreasing proportion of patients with treatment delay ≥60 days (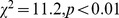, Figure 1C).

**Table 1:** Basic characteristics of the individuals at presentation.

	Number (%) patients						p-value
Group	Total	Physical examination	Delay<14 days	Delay 14–29 days	Delay 30– 59days	Delay≥60 days	
**Total**	48441	2261	18851	7811	8360	11053	0.000
**Patients 0–14 years of age**	151(0.3)	2(0.1)	58(0.3)	31(0.4)	28(0.3)	30(0.3)	0.000
**Patients 15–24 year of age**	16111(33.3)	843(37.3)	6490(34.4)	2596(33.2)	2699(32.3)	3436(31.1)	0.000
**Patients 25–44 year of age**	26792(55.3)	1262(55.8)	10553(56.0)	4256(54.5)	4640(55.5)	6037(54.6)	0.000
**Patients 45–64 year of age**	4053(8.0)	126(5.6)	1365(7.2)	695(8.9)	724(8.7)	1133(10.2)	0.000
**Patients ≥65 years of age**	1334(2.7)	28(1.2)	385(2.1)	233(3.0)	269(3.2)	417(3.8)	0.000
**Male patients**	30252(63.0)	1490(65.9)	12254(65.0)	4948(63.3)	5014(60.1)	6489(58.9)	0.000
**Migrant patients** *	37820 (78.1)	1762(77.9)	14910(79.0)	6038(77.4)	6481(77.5)	8544(77.3)	0.000
**Cavity patients**	10311(21.3)	115(5.1)	2815(14.9)	1721(22.0)	2207(26.4)	3450(31.2)	0.000
**Only constitutional**	2791(5.8)	68(3.0)	1526(8.1)	427(5.5)	323(3.9)	441(4.0)	0.000
**Only respiratory**	12839(26.5)	115(5.1)	4678(24.8)	2228(28.5)	2592(31.0)	3220(29.1)	0.000
**Both**	18214(37.6)	74(3.3)	4939(26.2)	3510(44.9)	4091(48.9)	5595(50.6)	0.000
**Clinical symptoms patients**	33844(69.9)	257(11.4)	11143(59.1)	6165(78.9)	7006(83.8)	9256(83.7)	0.000
**Sputum smear positive patients**	29836(61.6)	692(30.6)	9731(51.6)	4780(61.2)	2207(71.3)	8638(78.2)	0.000

* Migrants: people who reside Shenzhen but keep “*hukou*” in their hometown [12].

In a 3-level multivariate logistic regression analysis, compared with patients identified by routine physical examination, the risk of patients presenting with smear positive tuberculosis was 2.6 fold (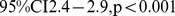) greater for delay <14 days, 3.9 fold (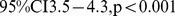) greater for delay 14–29 days, 5.4 fold (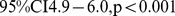) greater for delay30–59 days, and 7.6 fold (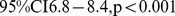) greater for delay ≥60 days (Table 2). Similarly, patients were 2.2 fold (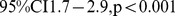), 3.3 fold (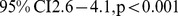), 4.0 fold (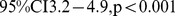), and 5.0 fold (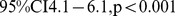) more likely to present with a pulmonary cavity with delays of <14 days, 14–29 days,30–59 days, and ≥60 days, respectively, compared to those identified on routine physical examination (Table 2).

**Table 2:** Effect of delay on transmission of tuberculosis.

	Sputum smear positive		Cavity	
	Adjusted OR (95%CI)	p-value	Adjusted OR (95%CI)	p-value
**Fixed effect**				
** Constant**	0.28 (0.17, 0.46)	<0.001	0.13 (0.08, 0.20)	<0.001
** Age (years)**				
≤14 year	1.00		1.00	
15–44 year	1.30 (0.89, 1.90)	0.175	0.97 (0.65, 1.44)	0.865
45–64 year	1.40 (0.96, 2.06)	0.084	1.14 (0.76, 1.70)	0.527
≥65 year	1.51 (1.01, 2.24)	<0.05	0.96 (0.63, 1.46)	0.840
** Sex**				
Men	1.00		1.00	
Women	1.07 (1.03, 1.11)	<0.005	0.73 (0.69, 0.77)	<0.001
** Migrant**				
No	1.00		1.00	
Yes	1.17 (1.11, 1.23)	<0.001	0.96 (0.89, 1.04)	0.351
** Treatment delay (days)**				
Physical examination	1.00		1.00	
Delay<14 days	2.60(2.35, 2.87)	<0.001	2.22(1.71, 2.90)	<0.001
Delay14–29 days	3.90(3.50, 4.34)	<0.001	3.29(2.66, 4.06)	<0.001
Delay 30–59 days	5.42(4.87, 6.04)	<0.001	3.99(3.23, 4.92)	<0.001
Delay ≥60 days	7.58 (6.82, 8.43)	<0.001	4.98(4.05, 6.13)	<0.001
** Random effect**				
Level 3-region	1.11(0.95, 1.30)	0.167	1.00(0.94, 1.06)	0.200
Level 2-year	1.60(1.40, 1.84)	<0.001	1.15(1.10, 1.20)	<0.001
Level 1-individual	e	–	e	–

In a multivariate regression analysis of risk factors associated with treatment delay, women (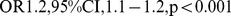) were associated with longer treatment delay (Table 3). Those who presented with constitutional symptoms alone were diagnosed and started on treatment earlier than those with respiratory symptoms alone (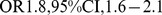) or with both respiratory and constitutional symptoms (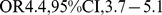), suggesting that respiratory symptoms are often misdiagnosed, delaying appropriate TB treatment (Table 3).

**Table 3:** Risk factors related with treatment delay.

Group	Mean(days)	Adjusted OR (95%CI)	p-value
**Total**	18		
**Constant**	–	5.35(2.31, 12.34)	<0.001
**Region**	–	1.28(0.89, 1.83)	0.171
**Year**	–	0.91(0.91, 0.92)	<0.001
**Age**	–		
≤14 year	18	1.00	
15–44 year	17	0.79(0.46, 1.35)	0.860
45–64 year	25	1.22(0.71, 2.12)	0.593
≥65 year	30	0.84(0.73, 2.31)	0.631
**Sex**			
Male	16	1.00	
Female	22	1.16(1.08, 1.22)	<0.001
**Migrant**			
No	20	1.00	
Yes	18	0.95(0.56, 1.64)	0.318
**Type of symptoms**			
Only constitutional	10	1.00	
Only respiratory	24	1.84(1.59, 2.13)	<0.001
Both	31	4.39(3.74, 5.13)	<0.001

## Discussion

In our retrospective study of 48,441pulmonary TB patients in Shenzhen, China from 1993–2010, 95.3% of TB patients self presented for medical evaluation after a median 18 days of illness. The remaining 4.7% of patients were diagnosed with TB during a routine physical examination. Overall, 61.6% presented sputum smear positive and 21.3% presented with a pulmonary cavity on chest x-ray, with the proportion sputum smear positive and pulmonary cavity positive both increasing significantly with increasing duration of treatment delay. Those with delays ≥60 days were 7.6 times (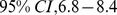) more likely to be sputum smear positive and 5.0 times (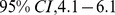) more likely to have a pulmonary cavity compared to those identified with TB during a routine physical examination. Because being sputum smear positive and having a pulmonary cavity are associated with a higher risk of TB transmission [20], [21], reducing treatment delay would thereby reduce the risk of TB transmission.

Many studies in multiple countries have assessed the factors associated with diagnostic and treatment delays, with the associated factors primarily related to patients or the healthcare system [22]–[28]. One common factor for delay across many of the studies was the lack of sufficient awareness of the symptoms and signs of TB among the general public and healthcare providers, causing either a delay in presentation or in diagnosis. In our analysis, due to its retrospective nature, we were unfortunately not able to analyze the specific reasons for delay. Instead, we analyzed the effect of the duration of treatment delay on the proportion sputum smear positive and pulmonary cavity positive. We used these two measures as a proxy for TB transmission risk because they have both been associated with TB transmission [20], [21]. Our analysis demonstrates the significantly increased risk of sputum smear positivity and proportion developing a pulmonary cavity with increasing duration of treatment delay, with those delaying more than 2 months having a 7–8 fold increased risk (Table 2). Additional studies are needed to understand not just how to incorporate individual interventions to reduce disease transmission, such as conducting physical examinations of migrants, patient and provider education, improved diagnostic tests, and active case finding, but how to incorporate a multi-factorial, combination strategy that not only seeks to reduce disease transmission but also aims to reduce disease progression from latent to active disease [29]. Both of these types of strategies will likely be needed to ultimately control the TB epidemic.

The duration of treatment delay in Shenzhen gradually decreased from 1993–2010, particularly among those with delays ≥60 days (Figure 1B), which is consistent with the global trend [30]. Although we were not able to identify specific reasons for this trend in our analysis, Shenzhen began providing free treatment for all TB patients in 2002 [16] and has systematically improved its overall TB control strategies [17], [18], especially for migrants [16], [18]. These policies may have played a significant role in reducing diagnostic and treatment delay. Our study identified no significant variance in treatment delay duration between migrants and non-migrants in Shenzhen. Although this is different from previous studies elsewhere in China [31], [32], other cities also do not consist of 80% migrants [14] and have not provided a similar level of support services to migrants as Shenzhen.

Our work is subject to certain limitations. First, the duration of treatment delay may be influenced by recall bias and may not be exactly accurate. However, this should not affect the overall trend and should also be the same for all patients, thus would not affect our finding an association between sputum smear positivity and having a pulmonary cavity with increasing duration of treatment delay. Second, we were not able to measure actual disease transmission and thus used two proxy measures. However, these two measures have been are well associated with TB transmission and have been used by previous studies [20], [21]. Finally, we were not able to identify specific factors, such as policy change, related to treatment delay from our data due to its retrospective nature. However, this question has been studied repeatedly by other groups and we have no reason to believe that treatment delay factors in Shenzhen would be any different.

Delays in TB diagnosis and treatment carry significant risks of worsening disease severity to the patient and increased risk of transmission to those around him/her. The challenge is to determine how to reduce this duration of delay, particularly in resource-limited settings where rapid diagnostic techniques may not be available or affordable. Shenzhen has done a commendable job already of reducing treatment delay duration ≥60 days and further studies are needed to understand the specific factors that contributed to this decrease.
